# Asgard archaea: Diversity, function, and evolutionary implications in a range of microbiomes

**DOI:** 10.3934/microbiol.2019.1.48

**Published:** 2019-01-30

**Authors:** Fraser MacLeod, Gareth S. Kindler, Hon Lun Wong, Ray Chen, Brendan P. Burns

**Affiliations:** 1 School of Biotechnology and Biomolecular Sciences, The University of New South Wales, Sydney, Australia; 2 Australian Centre for Astrobiology, The University of New South Wales, Sydney, Australia

**Keywords:** Asgard, archaea, eukarya, ecology, evolution, eocyte, genome, microbiome, phylogeny, metabolism

## Abstract

Elucidating the diversity of the Archaea has many important ecological and evolutionary implications. The Asgard superphylum of the archaea, described recently from metagenomic data, has reignited the decades-old debate surrounding the topology of the tree of life. This review synthesizes recent findings through publicly available genomes and literature to describe the current ecological and evolutionary significance of the Asgard superphylum. Asgard archaea have been found in a diverse range of microbiomes across the globe, primarily from sedimentary environments. Within these environments, positive correlations between specific members of the Asgard archaea and Candidate Division TA06 bacteria have been observed, opening up the possibility of symbiotic interactions between the groupings. Asgard archaeal genomes encode functionally diverse metabolic pathways, including the Wood-Ljungdahl pathway as a carbon-fixation strategy, putative nucleotide salvaging pathways, and novel mechanisms of phototrophy including new rhodopsins. Asgard archaea also appear to be active in nitrogen cycling. Asgard archaea encode genes involved in both dissimilatory nitrate reduction and denitrification, and for the potential to use atmospheric nitrogen or nitrite as nitrogen sources. Asgard archaea also may be involved in the transformation of sulfur compounds, indicating a putative role in sulfur cycling. To date, all Asgard archaeal genomes identified were described as obligately anaerobic. The Asgard archaea also appear to have important evolutionary implications. The presence of eukaryotic signature proteins and the affiliation of Asgard archaea in phylogenetic analyses appears to support two-domain topologies of the tree of life with eukaryotes emerging from within the domain of archaea, as opposed to the eukaryotes being a separate domain of life. Thus far, Heimdallarchaeota appears as the closest archaeal relative of eukaryotes.

## 1. Introduction

The Archaea constitute a significant portion of the Earth's microbial diversity, being dominant members of marine and soil environments [1]–[3]. With the emergence of cultivation-independent techniques, our understanding of the diversity of the Archaea is increasing, as highlighted by recent reviews [4],[5]. The diverse domain of archaea is currently divided into the superphyla of Euryarchaeota, TACK (Thaumarchaeota, Aigarchaeota, Crenarchaeota and Korarchaeota), DPANN (Diapherotrites, Parvarchaeota, Aenigmarchaeota, Nanoarchaeota, Nanohaloarchaeota, Woesearchaeota, Pacearchaeota and potentially Altiarchaea) and the recently described Asgards, consisting of Lokiarchaeota, Thorarchaeota, Odinarchaeota and Heimdallarchaeota. Members of the Asgard archaea have been described from a diverse range of habitats, including hydrothermal sediments [6]–[8], microbial mats [9], as well as a range of freshwater [10] and marine [8],[11]–[13] environments.

Thus far, a small number of studies have discussed the metabolic pathways of Asgard archaea. Some members of the Asgard archaea have been described as mixotrophic, with putatively important roles in sulfur and nitrogen cycling [11],[12]. Also identified in the genomes of Asgard archaea are a new class of rhodopsin, suggesting a phototrophic lifestyle for the Asgard [14]. Arsenic and copper resistance genes have also been identified within the genomes of Asgard archaea [9]. While a description of the metabolisms of Asgard archaea in the literature is limited, publicly available genomes are rich sources of information for reconstructing Asgard archaeal metabolic pathways.

The identification of Asgard archaea from metagenomic data has had important implications for our understanding of evolution. Historically, life has been divided into the domains of bacteria, eukarya and archaea, with the archaea and eukarya described as sister groups (referred to as the three-domain tree of life) [15]–[17]. Conversely to the three-domain topology of the tree of life, the Eocyte hypothesis proposes that the eukarya emerged from within the domain of archaea (the tree of life consisting of just the domains of archaea and bacteria), which appeared to be supported by the similarities in protein compositions between eukaryotes and a specific phylum of archaea: the Crenarchaeota [18],[19]. More recently, archaea of the TACK superphylum were found to contain proteins related to those in eukaryotes and to affiliate with eukaryotes in phylogenetic analysis, as discussed in recent reviews [20]. The Asgard archaea have increased the strength of the eukaryotic-archaeal affiliation in phylogenetic analyses [6],[8]. The Asgard archaea were also found to encode genes involved in numerous eukaryotic characteristic systems, many of which were formerly considered to be eukaryotic-specific [6],[8].

Here we review the latest discoveries in relation to the Asgard superphylum, including the controversies surrounding their discovery. Specifically, this review will discuss how cultivation-independent sequencing data has informed our understanding of the ecology, lifestyle, and evolutionary implications of the Asgard superphylum.

## 2. Asgard ecology

### 2.1. Distribution of Asgard archaea

**Figure 1.**
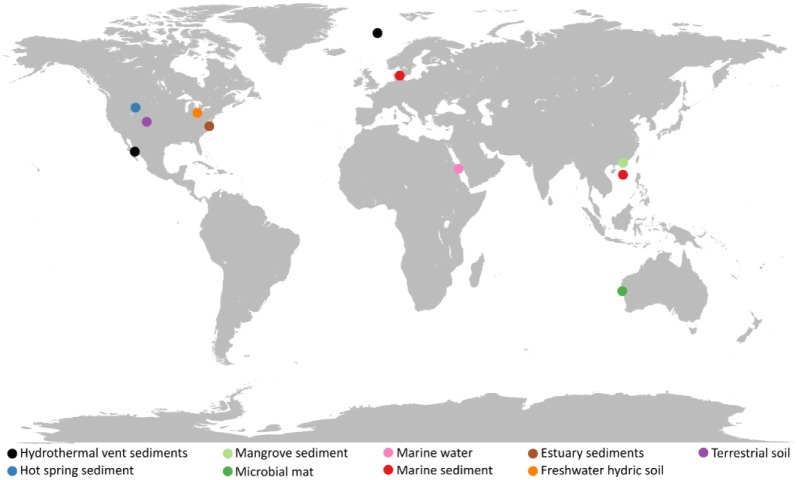
Global distribution of metagenomic-assembled sequences of Asgard archaea. Asgard metagenomic-assembled genomes from NCBI Assembly and MG-RAST databases were recorded for information related to location and environmental context of sampling (November 2018) (Table S1).

Asgard archaea were formerly grouped as Marine Benthic Group B (MBG-B) [21], Ancient Archaeal Group (AAG) [22], Deep-Sea Archaeal Group (DSAG) [23],[24], and Marine Hydrothermal Vent Group (MHVG) [22] archaea. Lokiarchaeota, the first phylum of Asgard archaea to be described, was initially identified from deep marine sediments of a hydrothermally active site [6]. Later, the Asgard archaeal phyla of Thorarchaeota [11], Odinarchaetoa, and Heimdallarchaetoa [8] were characterized by metagenomic profiling of a range of sedimentary environments across the globe. The habitats of which Asgard archaea have been metagenomically characterized from are largely anaerobic, including deep marine sediments, terrestrial soils and pelagic water (Figure 1).

Lokiarchaeota appear more abundant in currently characterized microbiomes, whereas Thorarchaeota, Heimdallarchaeota, and Odinarchaetoa appear at relatively lower abundances [8]. Environmental information acquired from NCBI and MG-RAST metagenomic databases (Table S1) associate Odinarchaeota with geothermal environments [8]. Heimdallarchaeota and Lokiarchaeota are primarily found in marine sediments whereas Thorarchaeota appears to occur in a diverse range of microbiomes, notably, marine, lake, and estuarine sediments (Table S1) [8]. Overall, Asgard archaea are more abundant in methane-rich or hydrothermal environments, rather than terrestrial soil or freshwater environments. The analysis of high-throughput 16S rRNA data may provide further insights into the distribution of Asgard archaea. Current and future research should aim to distinguish Asgard archaeal 16S rRNA operational taxonomic units (OTUs) to uncover more information regarding their habitats.

### 2.2. Associations with other microbial groups

Asgard archaea may play key roles in the function of ecosystems through microbe-microbe interactions. Correlations between microorganisms whether positive or negative can provide insight into interactions such as signaling systems, metabolite exchange, and other biotic or physiochemical activities [25]. Deducing the microbial relationships from correlation network studies can help determine the drivers behind Asgard archaea function and how habitat niches form and operate [26]. A recent study observed a strong positive correlation between Lokiarchaeota and the bacterial division TA06 within the microbial mats of Salar de Llamara, Chile; a warm, saline and shallow marine environment [27]. The abundance of Lokiarchaeota and TA06 was relatively low in the deeper sections of the microbial mats yet they exhibited the only strong positive correlation of Asgard archaea in this system [27]. Both TA06 and Lokiarchaeota are known to live in anoxic conditions and therefore this correlation may pose an interesting bacterial-archaeal interaction that requires further study. Co-occurrence analysis of Asgard groupings with other microbes may offer insights into potential mutualistic interactions. The correlations and specific interactions observed so far are unexplained, offering an opportunity to bridge the gap in knowledge. Among other microorganisms, Asgard archaea are mostly exposed to anoxic conditions as well as a range of elements such as organic and inorganic carbon sources, sulfur, and nitrogen [28]. These conditions provide the substrates for their metabolism and multiple studies have begun to uncover their metabolic capabilities, reviewed in the following section.

## 3. Metabolisms of Asgard archaea

### 3.1. Carbon metabolism

**Figure 2.**
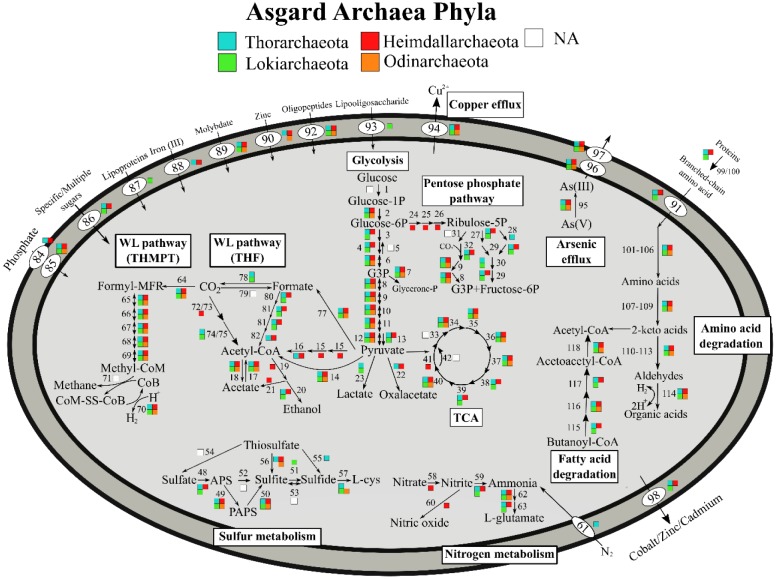
Metabolic pathways of Asgard archaea indicating variation at the phyla level. Carbon metabolic pathways indicate a heterotrophic lifestyle for Asgard archaea. Asgard archaea also encoded genes related to sulfur and nitrogen cycling.

**Figure 3.**
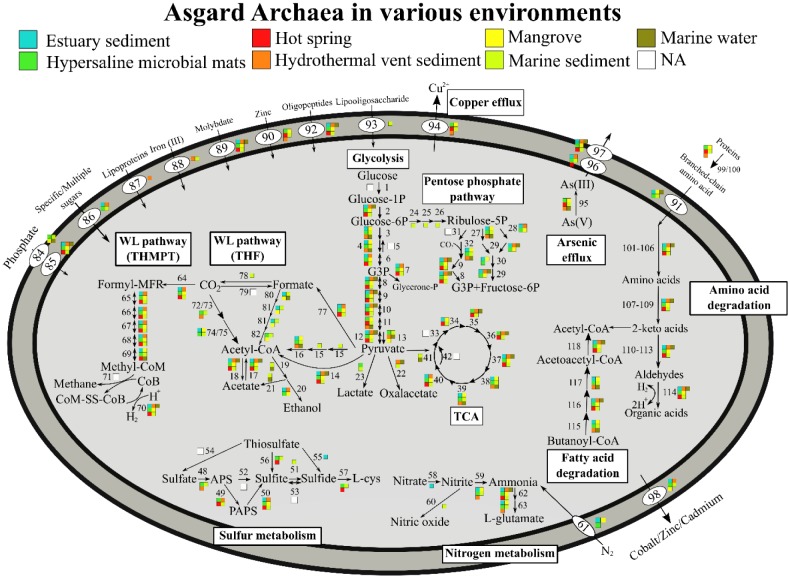
Differences in metabolic capabilities of Asgard archaea from different environmental samples. No significant variation was observed for Asgard archaea sampled from different environments. This may be due to the similarities of the sedimentary environments from which Asgard archaea were sampled.

Glycolysis appears to be ubiquitous among Asgard archaea, with all the genomes only lacking hexokinase (Figure 2). All four phlya have the genetic capacity to degrade peptides, while all but Odinarchaeota encodes for a complete butanoyl-CoA oxidation pathway. Since all Asgard archaea are able to obtain carbon sources through degrading carbohydrate and peptides, this indicates a heterotrophic lifestyle [11],[12],[29] (Figures 2 and 3). A summary of putative metabolic genes identified is given in Table S2.

Wood-Ljungdahl (WL) pathways were also identified in Asgard archaea [9],[12],[29]. There are two types of WL pathway, one using tetrahydrofolate (THF) as the C_1_ carrier while the other one uses tetrahydromethanopterin (THMPT). Generally, bacteria encode for the THF-WL pathway while archaea harbor the THMPT-WL pathway [30],[31]. Studies have identified that Thorarchaeota from sulfate-methane transition zones, hypersaline microbial mats, and mangroves harbor a complete set of genes in the THMPT-WL pathway, suggesting the ability to fix carbon dioxide through autotrophic acetogenesis [9],[11],[12]. These Thorarchaeota genomes also encode for genes associated with the THF-WL pathway, indicating they can use both THMPT and THF as a C_1_ carrier [9],[12].

On the other hand, Lokiarchaeota was shown to be a hydrogen-dependent, anaerobic autotrophic archaeon, harboring a complete THMPT-WL pathway, which would facilitate the fixing of carbon dioxide [29]. In the present study 23 Asgard archaeal genomes were downloaded from public repositories and annotated against the KEGG database [32]. Despite being previously unreported, we found that both Heimdallarchaeota and Odinarchaeota also encode a complete THMPT-WL pathway, with Heimdallarcheota also encoding for a complete THF-WL pathway (Figure 2). Given the fact that Asgard archaea are capable of metabolizing autotrophically and heterotrophically, this suggests that this superphylum lives a mixotrophic lifestyle, although such a lifestyle was only reported in Thorarchaeota [12]. To summarize, all four Asgard archaea phyla putatively utilize THMPT as C_1_ carriers while both Thorarchaeota and Heimdallarchaeota can also use THF as C_1_ carriers (Figure 2) [33]. As the Wood-Ljungdahl pathway is the only complete carbon fixation pathway identified in the available data, it is likely that Wood-Ljungdahl pathway is the preferred carbon fixation pathway among the Asgard archaea.

Other partially complete carbon fixation pathways such as the 4-hydroxybutyrate cycle were also found in Thorarchaeota and Lokiarchaeota, with the genomes encoding for the key genes 4-hydroxybutytyl-CoA dehydratase and enoyl-CoA hydratase. Although only previously reported in Asgard archaea genomes from hypersaline microbial mats [9], the aforementioned genes were also identified in Asgard genomes from marine sediments, estuary sediments and mangroves [8],[11],[12]. Interestingly, the gene responsible for reverse TCA (rTCA) cycle was found in two Lokiarchaeota genomes from marine sediment and hypothermal vent sediment respectively, which has not been previously reported.

Thorarchaeota, Lokiarchaeota, and Heimdallarchaeota were found to harbor ribulose bisphosphate carboxylase (RuBisCo), the main gene in the carbon-fixing Calvin-Benson-Bassham phototrophic pathway [9],[11],[12]. However, phylogenetic analysis revealed that the Asgard archaeal RuBisCo are not photosynthetic, but rather affiliated with type IV or archaeal type III. These forms of RuBisCo are not involved in the Calvin-Benson-Bassham (CBB) cycle, but rather in salvaging and assimilating nucleosides [34],[35]. This infers that Asgard archaea is capable of assimilating salvaged nucleotides into central carbohydrate pathways.

Recently, a type 1 proton-pumping rhodopsin was identified in Heimdallarchaeota and Thorarchaeota, which was coined as a novel pigment—heliorhodopsin [14]. This suggests that in the early Earth, heliorhodopsin-bearing Asgard archaea were likely located at the top of light-exposed habitats. This highlights the potential ability of Asgard archaea to sense and capture sunlight.

### 3.2. Nitrogen and sulfur cycling

Evidence of nitrogen cycling potential was found in Heimdallarchaeota genomes, possessing both nitrate reductase and nitrite reductase (NO forming), suggesting its involvement in dissimilatory nitrate reduction (DNRA) and denitrification (Figure 2). On the other hand, nitrogenase was only reported in Thorarchaeota from hypersaline microbial mats and mangroves [9],[12], inferring their role in assimilating atmospheric nitrogen into ammonia. Nitrite reductase (NADH) was also identified in all Asgard archaeal phyla except Odinarchaeota, where it was suggested that nitrite could be the nitrogen source among Asgard archaea other than peptides [9],[12]. This also suggests that Asgard archaea play a role in nitrogen transformation.

All Asgard archaea encode for both sulfate adenylyltransferase and phosphoadenosine phosphosulfate reductase, indicating their putative ability in assimilatory sulfate reduction and sulfate activation (Figure 2). There were no studies reporting dissimilatory sulfate reduction and sulfur oxidation (SOX) systems. However, evidence of thiosulfate reductase and sulfhydrogenase were identified in Thorarchaeota in estuary sediments, while thiosulfate sulfurtransferase was identified in Thor-, Heimdall- and Odinarchaeota [8]. This suggests that these organisms may be involved in the transformation (particularly reduction) of intermediate sulfur compounds [11].

### 3.3. Arsenic metabolism and copper resistance

All four Asgard archaeal phyla have the genomic capacity to reduce As(V) with arsenate reductase and efflux arsenite through arsenite transporter (Figure 2). High arsenic concentrations were detected in mangrove sediments, however low levels were detected in hypersaline microbial mats [9],[36]. Arsenic transformation and resistance appear to be ubiquitous in various environments among Asgard archaea irrespective of arsenic concentrations (Figure 3), suggesting arsenic metabolisms as a relic of an ancient microbial trait [37],[38]. Similarly for copper resistance, despite only Shark Bay hypersaline microbial mats having been reported with relatively high copper concentrations [9], most of the genomes harbor a copper efflux system, inferring this as a potential universal trait among Asgard archaea in different microbiomes.

### 3.4. Anaerobic lifestyle

As most Asgard archaeal genomes and 16S rDNA to date were extracted from anoxic environments [8],[9],[11],[12],[27],[30], and most Asgard archaea lack a complete tricarboxylic acid cycle (TCA) cycle and are capable of anaerobic metabolisms such as WL-pathway, they were thought to be obligately anaerobic.

## 4. Evolutionary implications of the Asgard superphylum

**Figure 4.**
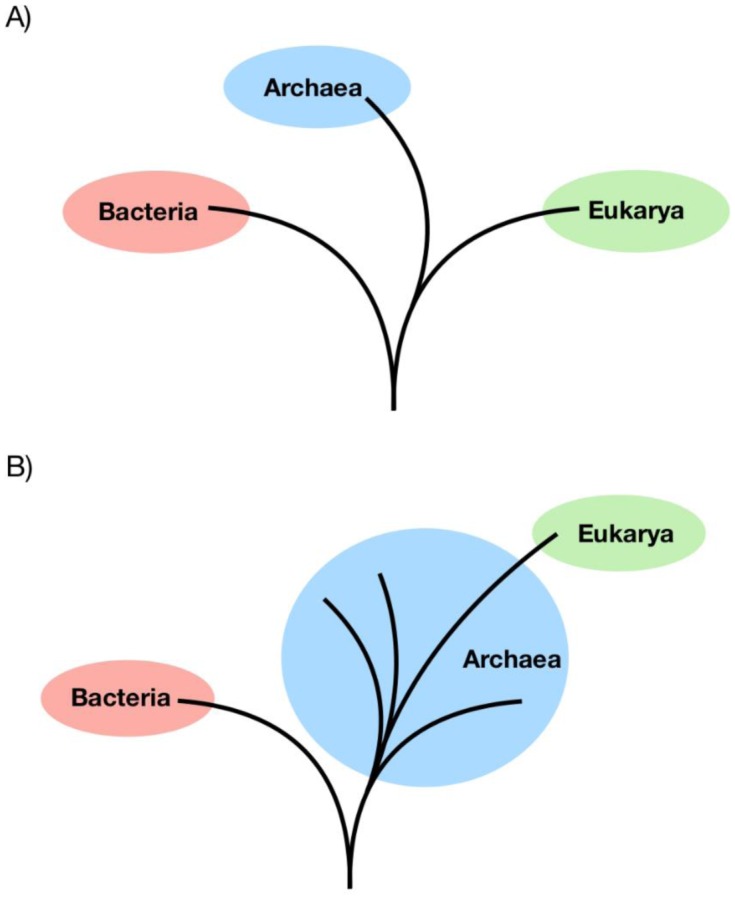
Placement of eukaryotes within the tree of life. The two competing hypotheses: (A) the Woese three domain tree of life, whereby eukaryotes share a common ancestor to the exclusion of the bacteria, (B) the Eocyte hypothesis, whereby eukaryotes emerged from within the diversity of archaea.

The origin of the eukaryotic cell has remained one of the most contentious debates in evolutionary biology. Two hypothesises have emerged to describe the evolution of the eukaryotic cell, depicted in Figure 4. Firstly, the Woese model of archaea and eukarya diverging from a common ancestor to the exclusion of bacteria [16], and secondly the Eocyte hypothesis, in which eukarya emerged from within the archaea [18]. The recent reporting of the Asgard superphylum has given support to the Eocyte hypothesis [6],[8], although this has added controversy within the context of the current Woese tree of life dogma, with alternative analyses of Asgard archaea supporting Woese models [39].

### 4.1. Evolutionary relationship between Asgard archaea and eukaryotes

**Figure 5.**
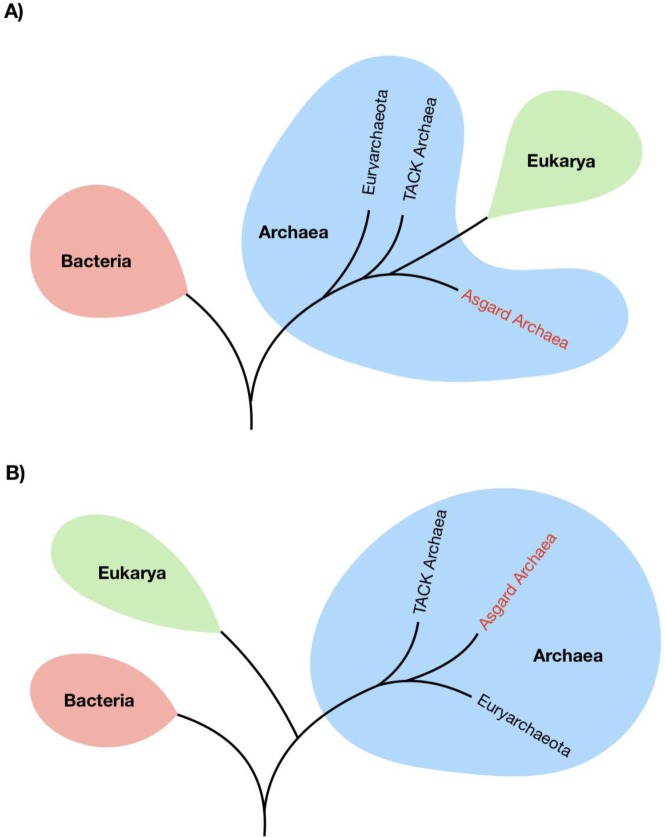
Differing phylogenetic trees resulting from the inclusion of the Asgard superphylum. (A) The tree of life resulting from a range of phylogenetic analyses of conserved markers, ribosomal RNA genes and ribosomal proteins, placing Asgard archaea as the closest archaeal relatives of eukaryotes. (B) The tree produced from phylogenetic analyses of Asgard RNA polymerase genes supports the three-domain topology of the tree of life, with Asgard archaea as a sister group to Euryarchaeota.

The identification of the Asgard superphylum from metagenomic data has been taken as evidence of the Eocyte hypothesis. In the original phylogenetic analyses of an Asgard archaea, Lokiarchaeota was found to represent a monophyletic clade of the TACK superphylum [6]. Inclusion of eukarya in such phylogenies positioned eukarya within the Asgard archaea, hinting at the close relationship between the two groups. At the time, it was suggested that Lokiarchaeota represented the closest archaeal relatives of eukaryotes [6]. The later identified phyla of Thorarchaeota [11], Heimdallarchaeota and Odinarchaeota [8], were found to form a monophyletic group with Lokiarchaeota, proposed as the Asgard superphylum, which represented a sister group to the TACK superphylum [8]. Phylogenetic analyses of the Asgard superphylum using ribosomal RNA genes, universally conserved marker genes and ribosomal proteins placed eukarya within the Asgard archaea, with Heimdallarchaeota suggested as the closest archaeal relatives of the eukarya (Figure 5A) [8],[12]. Although Asgard archaea affiliate strongly with eukarya in such phylogenetic analyses, the exact evolutionary relationship between the two groups has yet to be determined.

The placement of Asgard archaea as the closest archaeal relatives of eukaryotes has been criticized by some, which alternatively proposes the Asgard superphylum to be a sister group of the Euryarchaeota [39]. Da Cunha and colleagues [39] suggest that the placement of the eukaryotes within the Asgard superphylum or as a sister group to Asgard archaea in phylogenetic analyses is potentially the result of phylogenetic artefacts and/or contamination or homologous recombination of Asgard genomes with eukaryotic sequences. However direct evidence of contamination or recombination has yet to be shown, and thus further work is needed to definitively prove this claim. The accuracy of claims of contamination and/or homologous recombination have themselves been contested, with some claiming misinterpretation of the original findings and not accounting for the other previously published Asgard archaea (including Thor-, Heimdall- and Odinarchaeota) which also provide evidence of a strong eukaryotic affiliation [40]. As it is unlikely that contamination and/or homologous recombination with eukaryotic sequences occurred in the same manner across the Asgard archaea, genomes of which were assembled from a variety of sources using a variety of methodologies [6],[8],[12], it is unlikely that the eukaryotic-Asgard affiliation is the result of contamination with eukaryotic sequences.

Da Cunha and co-workers [39] performed their own phylogeny on RNA polymerases in the original Lokiarchaeota genome, developing alternative phylogenetic trees indicating Lokiarchaetoa is a sister group to the Euryarchaeota, supporting a three-domain tree of life (Figure 5B) [39]. However, the phylogenetic trees produced have been criticized for failing to account for phylogenetic artefacts and lack the statistical significance to draw strong conclusions about the placement of Asgard archaea in the tree of life [40].

### 4.2. Eukaryotic-like archaea

Genomic exploration found that the genome of Lokiarchaeota encoded numerous proteins that were previously considered to be eukaryotic specific [6]. Lokiarchaeota was found to possess eukaryotic signature proteins (ESP) involved in a broad range of eukaryotic process, including membrane-related processes and cell shape dynamics [6]. With the identification of Thorarchaeota, Odinarchaeota, and Heimdallarchaeota, the repertoire of ESP in Asgard genomes was expanded, suggesting that ESP are widespread across the Asgard superphylum. Thus far, Asgard archaea have been found to encode a diverse range of ESP, including proteins involved in eukaryotic membrane trafficking machinery, eukaryotic-like structural and cytoskeleton proteins, proteins involved the ubiquitination modification system, and eukaryotic-like ribosomal proteins [6],[8],[41]. The presence of eukaryotic-like systems in the Asgard superphylum suggests that eukaryotes inherited simple variations of eukaryotic cellular machinery from an archaeal ancestor. However, many of the eukaryotic systems putatively encoded in Asgard archaea are incomplete or are yet to be functionally characterized. As they possess yet unknown functions in archaea, it is difficult to infer the mechanism by which eukaryotic systems developed within the archaea.

While the majority of the ESP encoded in Asgard archaeal genomes are yet to be functionally annotated, recent research has begun to employ cultivation independent techniques to better understand how eukaryotic-like systems act in archaea. One of the key characteristics of eukaryotic cells is the dynamic cytoskeleton. Profilins are a known class of regulators of eukaryotic cytoskeleton dynamics [42]. It has been recently reported that Asgard archaea encode eukaryotic-like multifunctional profilins [6],[8],[43], unlike the singular function profilins of prokaryotes [44]. Although researchers have been unable to demonstrate their interaction with Asgard actins, Asgard profilins have been shown to interact with eukaryotic actin [43], despite the billions of years of evolution that have occurred since the divergence of the archaeal-eukaryotic common ancestor [45],[46]. The reporting of Asgard profilin which is both structurally and functionally related to its eukaryotic counterpart suggests that Asgard archaea have the potential for dynamic actin regulation [43], which could be explained by a common archaeal ancestor of eukaryotes and Asgard archaea.

Similarly, the formerly eukaryotic-specific ubiquitin modification system, which has been reported in a variety of archaea, was found to be functional in the uncultured archaeon *Candidatus* “*Caldiarchaeum subterraneum*” [47],[48]. Although not verified in Asgard archaea which also putatively encode components of a ubiquitin modification pathway [6],[8], such research indicates that the ubiquitin modification pathway may have eukaryotic-like functions in archaea. The presence of a functional, eukaryotic-like ubiquitination system in archaea suggests that eukaryotes inherited the ubiquitin modification system from an archaeal ancestor.

In addition to the abundance of ESP in Asgard genomes, some have noted the similarity between eukaryotic and Asgard archaeal RNA structures. The mechanisms by which the three domains of life insert selenocysteine (SEC) during translation are vastly different between the bacteria, the archaea, and the eukarya [49]–[51]. The selenocysteine insertion sequence (SECIS) of Lokiarchaeaota possesses similarity to the RNA structures of eukaryotic SECIS, as well as bearing similarity to the SECISs of archaeal selenoproteins [52]. Thus, it has been proposed that Lokiarchaeota encode an intermediate archaeal-eukaryotic selenocysteine insertion system, suggesting the eukaryotic SECIS has archaeal evolutionary origins [52].

## 5. Conclusions and future perspectives

This review has highlighted the state-of-the-art in relation to Asgard archaeal research, with a focus on key discoveries that have underpinned our understanding of the lifestyle, ecology, and evolution of this unique branch of life. Some of the discoveries are not without their controversies, however this leads to a healthy debate and more focused research directions that will benefit this field. As metagenomic, metatranscriptomic, and metaproteomic datasets from numerous environmental microbiomes continue to be resolved at rapid rates, it is likely novel aspects of this enigmatic group are waiting to be elucidated.
